# Molecular cloning and characterization of farnesyl diphosphate synthase from *Rosa rugosa* Thunb associated with salinity stress

**DOI:** 10.7717/peerj.16929

**Published:** 2024-02-29

**Authors:** Guo Wei, Yudie Chen, Jianwen Wang, Liguo Feng

**Affiliations:** College of Horticulture and Landscape Architecture, Yangzhou University, Yangzhou, China

**Keywords:** Rosa rugosa, Farnesyl diphosphate synthase, Terpenoids

## Abstract

*Rosa rugosa*, a renowned ornamental plant, is cultivated for its essential oil containing valuable monoterpenes, sesquiterpenes, and other compounds widely used in the floriculture industry. Farnesyl diphosphate synthase (FPPS) is a key enzyme involved in the biosynthesis of sesquiterpenes and triterpenes for abiotic or biotic stress. In this study, we successfully cloned and characterized a full-length FPPS- encoding cDNA identified as RrFPPS1 using RT-PCR from *R. rugosa*. Phylogenetic analysis showed that RrFPPS1 belonged to the angiosperm-FPPS clade. Transcriptomic and RT-qPCR analyses revealed that the *RrFPPS1* gene had tissue-specific expression patterns. Subcellular localization analysis using *Nicotiana benthamiana* leaves showed that RrFPPS1 was a cytoplasmic protein. *In vitro* enzymatic assays combined with GC-MS analysis showed that RrFPPS1 produced farnesyl diphosphate (FPP) using isopentenyl diphosphate (IPP) and dimethylallyl diphosphate (DMAPP) as substrates to provide a precursor for sesquiterpene and triterpene biosynthesis in the plant. Additionally, our research found that RrFPPS1 was upregulated under salt treatment. These substantial findings contribute to an improved understanding of terpene biosynthesis in *R. rugosa* and open new opportunities for advancements in horticultural practices and fragrance industries by overexpression of the *RrFPPS1* gene *in vivo* increased FPP production and subsequently led to elevated sesquiterpene yields in the future. The knowledge gained from this study can potentially lead to the development of enhanced varieties of *R. rugosa* with improved aroma, medicinal properties, and resilience to environmental stressors.

## Introduction

Terpenoids are the largest and most diverse groups of natural products, with over 80,000 structures identified to date (Christianson, 2017). They play various physiological and ecological roles that are essential for plant growth and development (Dudareva, 2007; Zhou & Pichersky, 2020) and are involved in various physiological processes such as hormone biosynthesis, photosynthesis, electron transport, and membrane components. Moreover, terpenoids act as important signaling and defense mediators between plants and their environments (Vandermoten, Haubruge & Cusson, 2009). Owing to their remarkable properties, these compounds have significant applications in the food, pharmaceutical, and agricultural industries, making them highly valuable resources (Holstein & Hohl, 2004).

In plants, terpenoids are synthesized from two independent pathways: the methylerythritol phosphate pathway (MEP) pathway in plastids (Lichtenthaler et al., 1997; Rodriguez-Concepcion & Boronat, 2002) and the mevalonate (MVA) pathway in cytosol or peroxisomes (Chen et al., 2011; Sapir-Mir et al., 2008). The MVA-pathway primarily supplies the precursor farnesyl diphosphate (FPP; C_15_) for the synthesis of sesquiterpenes, triterpenes and sterols (Valitova, Sulkarnayeva & Minibayeva, 2016; Natalia Dudareva et al., 2005). By contrast, the plastidic MEP-pathway provides the precursors such as geranyl diphosphate (GPP; C_10_), geranylgeranyl diphosphate (GGPP; C_20_), and geranylfarnesyl diphosphate (GFPP; C_25_) for the production of monoterpenes, diterpenes, and sesterterpenes, respectively (Chen et al., 2019; Chen et al., 2021; Arigoni et al., 1997; Huang et al., 2017; Schwender et al., 1997). These reactions are all catalyzed by enzymes commonly referred to as prenyltransferases. Both pathways can provide the terpene building blocks: isopentenyl diphosphate (IPP) and dimethylallyl diphosphate (DMAPP). According to the structure of enzymatic products, these prenyltransferases were grouped into geranyl diphosphate synthase (GPPS), farnesyl diphosphate synthase (FPPS), geranylgeranyl diphosphate synthase (GGPPS) and geranylfarnesyl diphosphate synthase (GFPPS), respectively (Chen et al., 2021; Cui et al., 2022; Song et al., 2023). They function at the branch points of isoprenoid metabolism and thus control IPP and DMAPP flux into different terpenoid families (Ogura & Koyama, 1998).

FPPS is a vital enzyme that catalyzes the head-to-tail condensation of IPP and DMAPP to produce FPP (Pichersky & Raguso, 2018). Numerous studies investigated the biochemical and molecular biology characteristics of FPPS in a variety of plant species, such as Arabidopsis, rice, maize, *Ginkgo biloba*, *Chimonanthus praecox*, and *Hedychium coronarium* (Cunillera, Boronat & Ferrer, 1997; Sanmiya et al. et al., 1997; Lan et al., 2013; Li & Larkins, 1996; Wang et al., 2004; Xiang, Zhao & Chen, 2010). In Arabidopsis, the *AtFPPS1* gene is primarily expressed in roots and flowers, and *AtFPPS2* is expressed mainly in flowers (Cunillera et al., 1996). In maize, FPPS1 plays a key role in the biosynthesis of FPP for ubiquinone production, and FPPS2 is primarily responsible for the biosynthesis of isofucosterol. Meanwhile, FPPS3 is closely linked to the production of sesquiterpene volatile (*E*)-*β*-caryophyllene in response to the presence of the root-chewing herbivore *Diabrotica virgifera* virgifera (Tang et al., 2022). Despite numerous studies regarding FPPS in various plants, data on FPP formation in *Rosa rugosa* are lacking*.*

*R. rugosa*, native to Eastern Asia, has been cultivated in the floriculture industry worldwide and is renowned for its essential oil extracted from petals, which is used in perfumes, cosmetics, and pharmaceuticals (Bendahmane et al., 2013; Caser & Scariot, 2022). *R. rugosa* ‘Zi Zhi’ is a continuously flowering variety that offers significant potential for yielding more essential oil (Bai et al., 2021). The essential oil of *R. rugosa* mainly consists of monoterpenes, sesquiterpenes and other valuable compounds (Ueyama et al., 1990). Despite significant knowledge about monoterpene biosynthesis, the molecular mechanisms of sesquiterpene biosynthesis in *R. rugosa* remain poorly understood. Given that FPPS plays a pivotal role in generating FPP, a critical precursor for sesquiterpene biosynthesis, unraveling the mechanisms of FPPS formation enhances our understanding of essential oil production in *R. rugosa.* Plant growth and development are often influenced by environmental stresses, with salinity stress being one of the most severe challenges (Zhu, 2001). Although FPPS is closely associated with terpenoid production, studies examining the effect of abiotic stress on FPPS, particularly in *R. rugosa*, are limited (Tian et al., 2018).

In this study, we aimed to identify potential FPPS from *R. rugosa*. A comprehensive characterization of the identified FPPS was then conducted to provide insight into the molecular mechanism of FPP formation. We also investigated whether the FPPS from *R. rugosa* exhibits any response to salt stress. Using a combination of bioinformatics, transcriptomic, molecular, and biochemical approaches, we successfully identified FPPS as the key enzyme responsible for FPP formation. Our findings address a gap in the molecular mechanism of sesquiterpene biosynthesis in *R. rugosa*.

## Materials & Methods

### Plant materials

Plant materials were collected from the resource nursery of Yangzhou University (32.391°N, 119.419°E), Yangzhou, China. The leaves and flowers of 3-year-old cutting seedlings of *R. rugosa* ‘ZiZhi’ were collected for further analysis (May/2022). The samples were immediately frozen in liquid nitrogen and then stored at − 80 °C for RNA extraction.

### RNA extraction and gene expression analysis

Total RNA was extracted from the collected plant tissues using the TaKaRa MiniBEST Plant RNA Extraction Kit (TaKaRa) as per the manufacturer’s instructions. The concentration and quality of the total RNA were evaluated through NanoDrop 1000 analysis and gel electrophoresis. For reverse transcription, first-strand cDNA was synthesized from the total RNA using a PrimeScript™ II 1st Strand cDNA Synthesis Kit (TaKaRa) following the manufacturer’s protocol. RT-qPCR analysis was performed with HiScript III RT SuperMix for qPCR (Vazyme) as per the manufacturer’s instructions.

The expression levels of RrFPPS in roots (OR: roots of open-air and TR: roots of tissue culture seedlings), branches (PB, SB, and TB represent the primary, secondary, and tertiary lateral branches, respectively) and flowers (S1–S7 represent the seven flower stages, respectively. S1, small bud stage; S2, large bud stage; S3, reddish stage; S4, flowering initiation stage; S5, initial opening stage; S6, semi-opening stage; S7, full opening stage) were analyzed by reverse transcription quantitative real-time PCR (RT-qPCR) and using the previous transcriptomic data (Wang et al., 2022) of *R. rugosa* ‘Zi Zhi’ providing the per kilobase of the exon model per million mapped fragments (FPKM) of *RrFPPSs*. ChamQ SYBR qPCR Master Mix (Vazyme) was used for RT-qPCR on a CFX96 real-time PCR platform (Bio-Rad). The internal control gene was *5.8S rRNA*. The primers for *RrFPPS* and the internal control gene were designed using the Genscript online website (https://www.genscript.com) and synthesized by Tsingke Biotech (Beijing). The primer sequences are provided in Table S1.

A 20 µL reaction mixture containing 1 µL of cDNA template, 10 µM of the primers, and 10 µL of 2 ×ChamQ SYBR qPCR Master Mix used in accordance with the manufacturer’s instructions. Reactions were performed with an initial incubation at 95 °C for 30 s, followed by 39 cycles at 95 °C for 10 s and 60 °C for 30 s. Three independent biological replicates with three technical replicates were prepared for each sample. The relative transcript levels of *RrFPPSs* in different tissues were calculated using the 2^−△△Ct^ method (Livak & Schmittgen, 2001) based on the threshold cycle (Ct) values generated from the CFX Manager software from Bio-Rad (Hercules, CA, USA). All the experiments were carried out at least in triplicate. Values were analyzed by ANOVA using GraphPad Prism (GraphPad Software, La Jolla, CA, USA). *P*-values less than 0.05 were considered significant.

### *In-silico* analysis of RrFPPS and phylogenetic analysis

Possible FPPS genes were searched using a well-characterized *Arabidopsis thaliana* FPPS (AAB07248.1) protein involved in the isoprenoid biosynthesis pathway as a query through BLASTP with the genome of *R. rugosa* ‘Zi Zhi’ (J Wang, L Feng, 2022, unpublished data) (Cunillera et al., 1996). Candidate genes were further confirmed by Pfam (http://pfam.xfam.org/) and the Conserved Domain Database. The physicochemical properties of RrFPPS were analyzed using the ProtParam tool from the online software Expasy (https://web.expasy.org/protparam/). Multiple alignments of amino acid sequences were conducted with CLUSTAL W and GENEDOC. Multiple sequence alignment of *RrFPPS* sequences and the construction of a phylogenetic tree, were achieved using the neighbor-joining method of MEGA X software (substitution type: amino acid and *p*-distance model) (Kumar et al., 2018). Bootstrapping was performed by resampling from the 1,000 replicates. Protein sequences used for alignment were as follows: *Arabidopsis thaliana* FPPS (At4g17190), *Rosa chinensis* G/FPPS1 (A0A2P6Q231), *Rosa chinensis* FPPS2 (A0A2P6QLH7), *Fragaria vesca* FPPS (XP_004294906), *Malus domestica* FPPS (AAM08927), *Centella asiatica* FPPS (AAV58896), *Lupinus albus* FPPS (P49351), *Humulus lupulus* FPPS (BAB40665), *Panax ginseng* FPPS (AAY87903), *Prunus persica* FPPS (XP_007211529), *Vitis vinifera* FPPS (AAX76910), *Potentilla anserina* FPPS (XP_050367607), *Hevea brasiliensis* FPPS (AAM98379), *Artemisia annua* FPPS (AAD17204), *Salvia miltiorrhiza* FPPS (AEZ55677), *Paeonia lactiflora* FPPS (AKJ26301), *Jasminum sambac* FPPS (AIY24422), *Ginkgo biloba* FPPS (AAR27053), and *Picea abies* FPPS (ACA21460). The GenBank accessions and protein sequences of FPPS are shown in Table S2.

### Isolation and cloning of the RrFPPS coding sequence

The open reading frame (ORF) of RrFPPS was amplified using PrimeSTAR Max DNA Polymerase (Takara) and PCR primers designed with NEB Tm Calculator (https://tmcalculator.neb.com/). Reverse-transcribed cDNA served as the PCR template. The PCR products were purified using the FastPure Gel DNA Extraction Mini Kit (Vazyme), cloned into pEASY-Blunt cloning vectors (TransGen Biotech), and transformed into *Escherichia coli* Trans1-T1 (TransGen Biotech) competent cells. The PCR-positive clones were selected and sequenced by Sangon Biotech (Shanghai, China). The sequence information of RrFPPS is shown in Table S2.

### Subcellular localization of RrFPPS proteins

The subcellular localization of RrFPPS was predicted using the bioinformatics analysis website WoLF PSORT (https://wolfpsort.hgc.jp/). The ORF of RrFPPS was amplified with specific primers, and inserted into the pCAMBIA 1300-35S-sGFP vector using the ClonExpress II One Step Cloning Kit (Vazyme) with Sac1/Xba1 restriction enzymes. The constructs and the empty vector (control) were transformed into Agrobacterium tumefaciens strain GV3101+P19 by the freezethaw method. Single positive Agrobacterium clones were grown in Luria-Bertani (LB) medium until the OD_600_ reached 0.5–0.6 and then infiltrated into 5 to 6 week-old *Nicotiana benthamiana* leaves. The fluorescence of the tobacco plant leaves was examined two days after infiltration at 488 nm using an LSM 880 confocal microscope (Zeiss) to obtain images of the GFP fluorescence signal.

### Purification of recombinant RrFPPS proteins and enzymatic assay

For enzymatic assays, RrFPPS was cloned downstream of the (His)6-tag sequences in pEASY Blunt E1 plasmids to express RrFPPS-His recombinant proteins. The constructed vectors were verified through DNA sequencing and transformed into chemically competent *E. coli* BL21 (DE3) pLysS cells (Transgen). Single positive colonies were selected and grown in LB medium containing ampicillin (100 µg/mL) at 37 °C in a shaking incubator until the OD_600_ of the culture reached 0.4–0.6. Afterward, 0.4 mM isopropyl *β*-D-thiogalactopyranoside (IPTG) was added, and the culture was incubated at 16 °C and 200 rpm for 12 h to induce the recombinant proteins. The induced cells were collected by centrifugation at 5,000 g for 10 min and stored at − 80 °C.

The cells were resuspended in binding buffer (20 mM Tris-HCl, 500 mM NaCl, 10% glycerol, 10 mM imidazole, and 0.5 mM phenylmethylsulfonyl fluoride (PMSF), pH 7.5) and sonicated to disrupt them. The lysate was centrifuged at 12,000 rpm for 30 min at 4 °C, and Ni- nickel-nitrilotriacetic acid (NTA) agarose (Sangon Biotech) was added to the supernatant for affinity purification following the manufacturer’s instructions. The protein was added to the desalting buffer (250 mM MOPS, 100 mM MgCl_2_, 50% glycerol (1:1, vol:vol), pH 7.5) to obtain desalted protein, which was then applied to a Sephadex Desalting Gravity Column (Sangon Biotech) in accordance with the manufacturer’s instructions.

Enzymatic activity assays were performed using gas chromatography–mass spectrometry (GC–MS) in a final volume of 100 µL reaction volume containing 66 µM IPP, 44 µM DMAPP, 10 µL desalted enzyme solution, and ddH_2_O up to 100 µL to determine the *in vitro* activity of RrFPPS. After incubation at 30 °C for 2 h, the assay mixture was hydrolyzed overnight at 30 °C using 1 unit of calf intestinal alkaline phosphatase (CIP) and 1 unit of apyrase from potatoes. The volatile products were adsorbed by headspace solid-phase microextraction using 30 µm (CAR/PDMS layer) and 50 µm (DVB layer) (Supelco Inc., Bellefonte, PA, USA) overnight. The volatile compounds were collected from headspace and subjected to GC–MS (Clarus SQ8T; PerkinElmer, Waltham, MA, USA). The experimental procedure involved initially heating to 50 °C and holding it for 1 min, then increasing to 120 °C at 5 °C min^−1^, 200 °C at 8 °C min^−1^, and 250 °C at 12 °C min^−1^ for 7 min. The mass spectrometry (MS) conditions included the emission current of 200 µA, ionization energy of 70 eV, and a mass scan range of 29–600 amu.

### Quantification of the transcript levels of *RrFPPS* genes under salt treatment

One-month-old *R. rugosa* plants were treated with 100 mM NaCl solution for 1 h, and their leaves were collected with three biological repetitions. Another three plants soaked in deionized water for 1 h were set as the control group (CK). The leaves of salt treatment and CK plants (three replications for each) were picked, immediately frozen with liquid nitrogen, and stored at −80 °C. For RNA isolation, and cDNA synthesis, the cDNA of salt treatment and control was used as the template for SYBR green PCR amplification to examine the RrFPPS expression pattern. ChamQ SYBR qPCR Master Mix (Vazyme) was used for RT-qPCR on a CFX96 real-time PCR platform (Bio-Rad), and the reactions were performed as previously reported (Livak & Schmittgen, 2001). Three independent biological replicates with three technical replicates were prepared for each sample. The relative transcript levels of RrFPPSs in different treatments were calculated using the 2^−△△Ct^ method (Livak & Schmittgen, 2001) based on the threshold cycle (Ct) values generated from the CFX Manager software (Bio-Rad, Hercules, CA, USA). The values were analyzed as previously mentioned.

## Results

### Identification of *RrFPPS* Genes in *R. rugosa* genome

To investigate terpene biosynthesis in *R. rugosa*, particularly terpenes derived from FPP. Analysis revealed two potential full-length *FPPS* genes designated as RrFPPS1 and RrFPPS2 (Rru05G067530 and Rru05G000330). The full-length cDNA sequence of RrFPPS1 measured 1,153 bp, including an 82 bp 5′-untranslated region and a 42 bp 3′-untranslated region. Meanwhile, the full-length cDNA sequence of RrFPPS2 was 1,449 bp, comprising a 104 bp 5′-untranslated region and a 316 bp 3′-untranslated region. The ORFs of RrFPPS1 and RrFPPS2 were each 1,029 bp in length, encoding a protein of 342 amino acids. The predicted molecular mass of the RrFPPS1 protein was 39.57 kDa with a theoretical pI of 5.39. Similarly, the predicted molecular mass of the RrFPPS2 protein was 39.42 kDa with a theoretical pI of 5.52.

**Figure 1:**
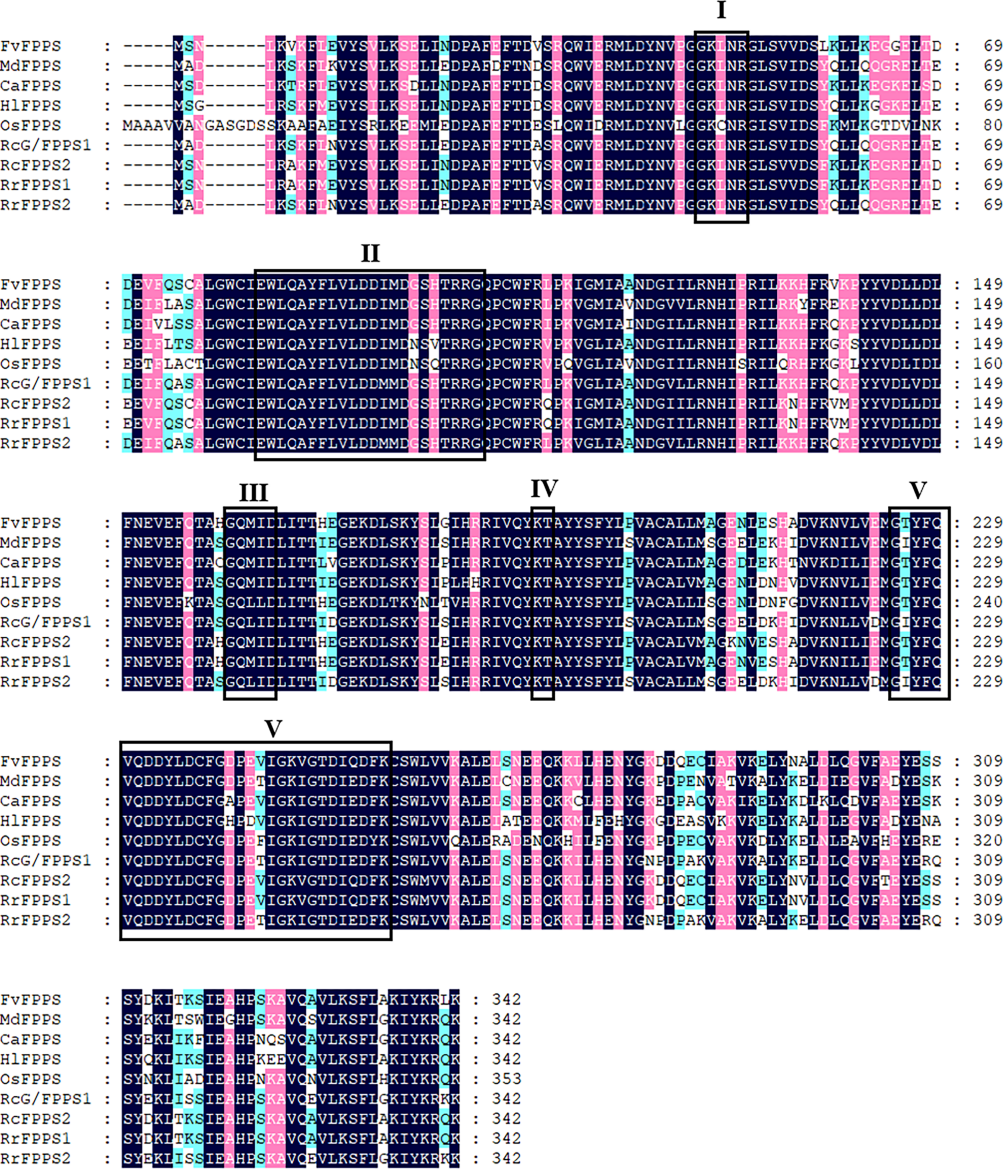
Sequence alignment of the deduced amino acid of *R. rugosa* FPPS and FPPSs from other plants. FvFPPS, *Fragaria vesca* FPPS; MdFPPS, *Malus domestica* FPPS; CaFPPS, *Centella asiatica* FPPS; HlFPPS, *Humulus lupulus* FPPS; OsFPPS, *Oryza sativa* FPPS; RcG/FPPS1, *Rosa chinensis* G/FPPS1; RrFPPS1, *Rosa rugosa* FPPS1; RrFPPS2, *Rosa rugosa* FPPS2. This alignment was shaded using Genedoc software to show conserved amino acid residues in dark blue and similar residues in pink and blue with a similarity of 75% and more than 50% respectively. The five conserved domains of prenyltransferases are boxed and marked by roman numerals (I–V), which are the substrate binding pocket, aspartate-rich regions 1, catalytic site, substrate-Mg^2+^ binding site and aspartate-rich regions 2. The highly conserved first and second aspartate rich regions (DD (XX)_2_-4D) are presented in domains II and V.

Sequence alignment analysis of RrFPPS protein sequences with other plant FPPS proteins revealed the presence of five highly conserved regions (I–V) that are essential for substrate binding and catalytic activityand typically seen in prenyltransferases (Fig. 1). These regions comprise the substrate binding pocket, substrate-Mg^2+^ binding site, catalytic site, and two aspartate-rich regions, namely, Asp-rich motif 1 and 2. The first is the first Asp-rich motif (FARM) in region II, with the sequence DD_XX(2-4)_D (D = aspartic acid and X = any amino acid), and the second is the second Asp-rich motif (SARM) in region V with the sequence DD_XX_D.

**Figure 2:**
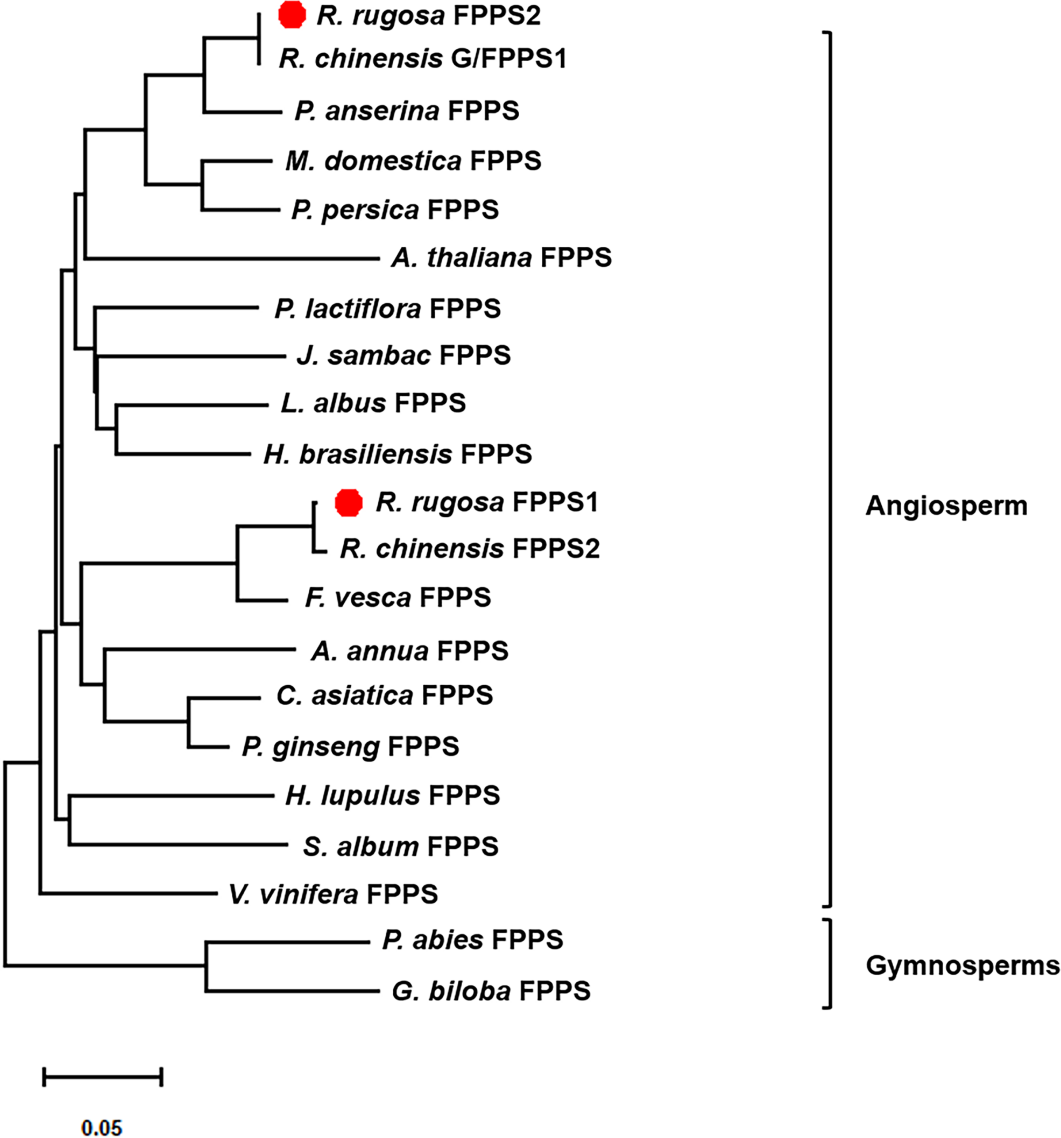
Phylogenetic tree of the amino acid sequences of FPPS from various organisms. The numbers in the figure represent the genetic distance. Bootstrap values are shown as a percentage of 1,000 replicates. RrFPPS1 and RrFPPS2 are marked by red dots. The scale bar on the bottom-left is representative of the degree of difference among sequences where a distance of 0.05 infers a 5% difference among sequences.

To investigate the evolutionary relationships between RrFPPS and FPPS proteins from other species, a phylogenetic tree was constructed. Phylogenetic analysis revealed that despite the high sequence homology, all plant FPPSs were separated into two major clades: clade of angiosperm and gymnosperm. Our analysis showed that RrFPPS have high homologous to FPPSs from other species. RrFPPS1 and RrFPPS2 appeared to fall in two separate subclades. Given that RrFPPS2 showed 100% identity to RcG/FPPS1 (*R. chinensis*), we focused on the biochemical function of RrFPPS1 hereafter (Fig. 2).

### Tissue expression profiling of the *RrFPPS* genes

**Figure 3:**
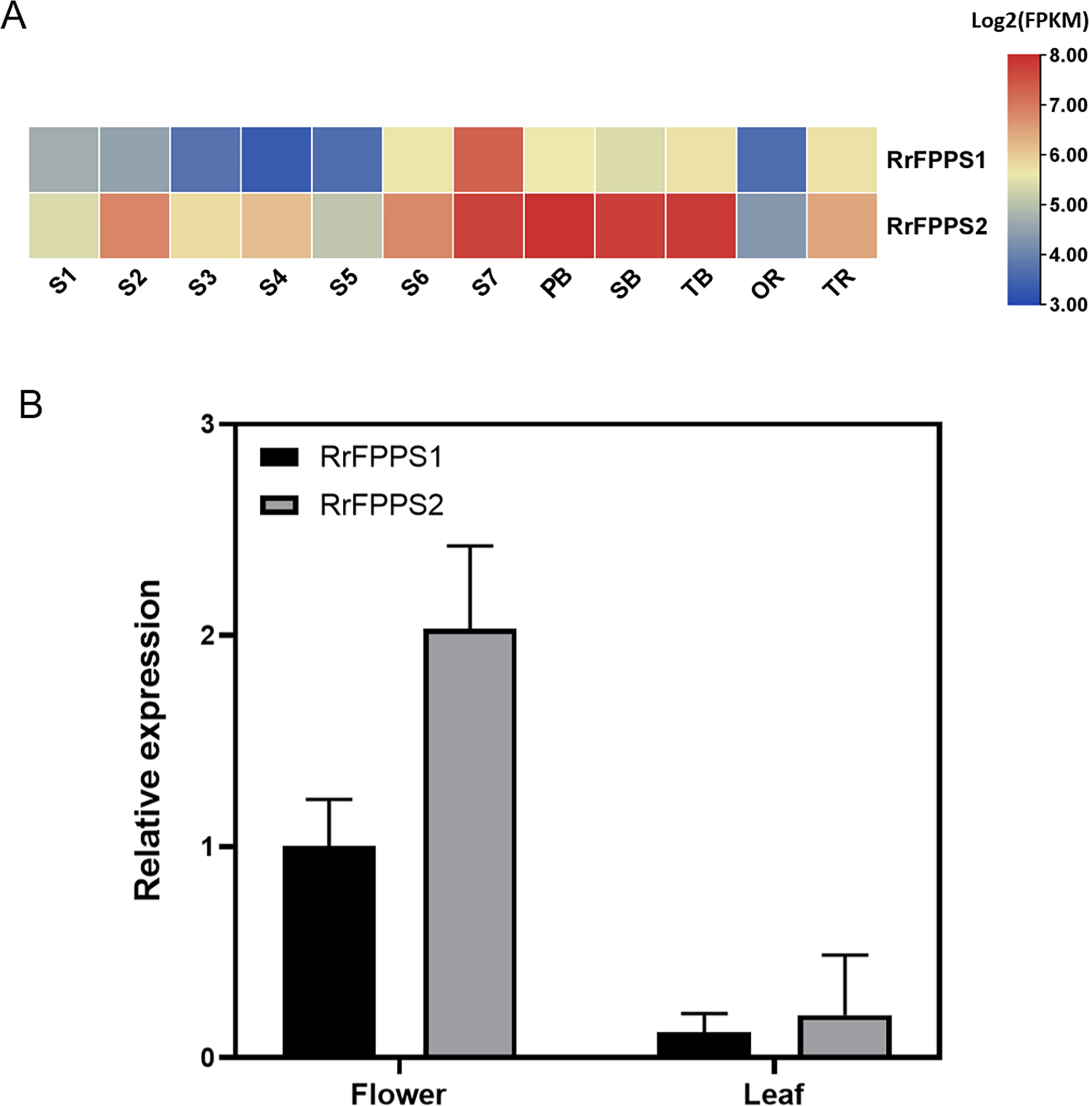
Analysis of gene expression of RrFPPS1 and RrFPPS2. (A) Gene expression profiles in the roots, lateral branches, and flowers of *R. rugosa* ‘Zi Zhi’. A heat map was generated based on the normalized Log2 FPKM represented by the blue-yellow gradation. The numbers in the heat map were FPKM from RNA-seq data. The OR and TR columns represent the roots of open-air and tissue culture seedlings, respectively The columns of FB, SB, and TB represent the primary, secondary, and tertiary lateral branches, respectively. The columns of S1–S7 represent the seven flower stages, respectively. (B) Gene expression of RrFPPS1 and RrFPPS2 in the flower and leaf of *R. rugosa* ‘Zi Zhi’ obtained with RT-qPCR. RrFPPS1 and RrFPPS2 expression values were normalized to the levels of 5.8S RNA expression in the respective stage. Data presented are 2^−ΔΔCt^ levels calculated relative to the special tissue. Data are presented as mean ± SE, *n* = 3.

To investigate the tissue-specific expression patterns of RrFPPS in *R. rugosa*, both transcriptomic analysis and RT-qPCR were used to evaluate and quantify transcript levels. Transcriptomic analysis showed that a high transcript of RrFPPS2 was detected in all tissues, and a high transcript of RrFFPPS1 was detected in S7 of the flower. In flowers, the expression level of RrFPPS2 was higher than that of RrFPPS1. Both genes had increased expression from S1 to S7, reaching the maximum level at the S7 stage. Moreover, RrFPPS2 had the highest transcript in branches (Fig. 3A). RT-qPCR results of leaves and flowers from 3-year-old cutting seedlings also revealed that RrFPPS1 and RrFPPS2 were similarly expressed in two tissues but with distinct expression patterns. Both genes showed higher relative expression levels in flowers than in leaves (Fig. 3B).

### Subcellular localization of the RrFPPS proteins

**Figure 4:**
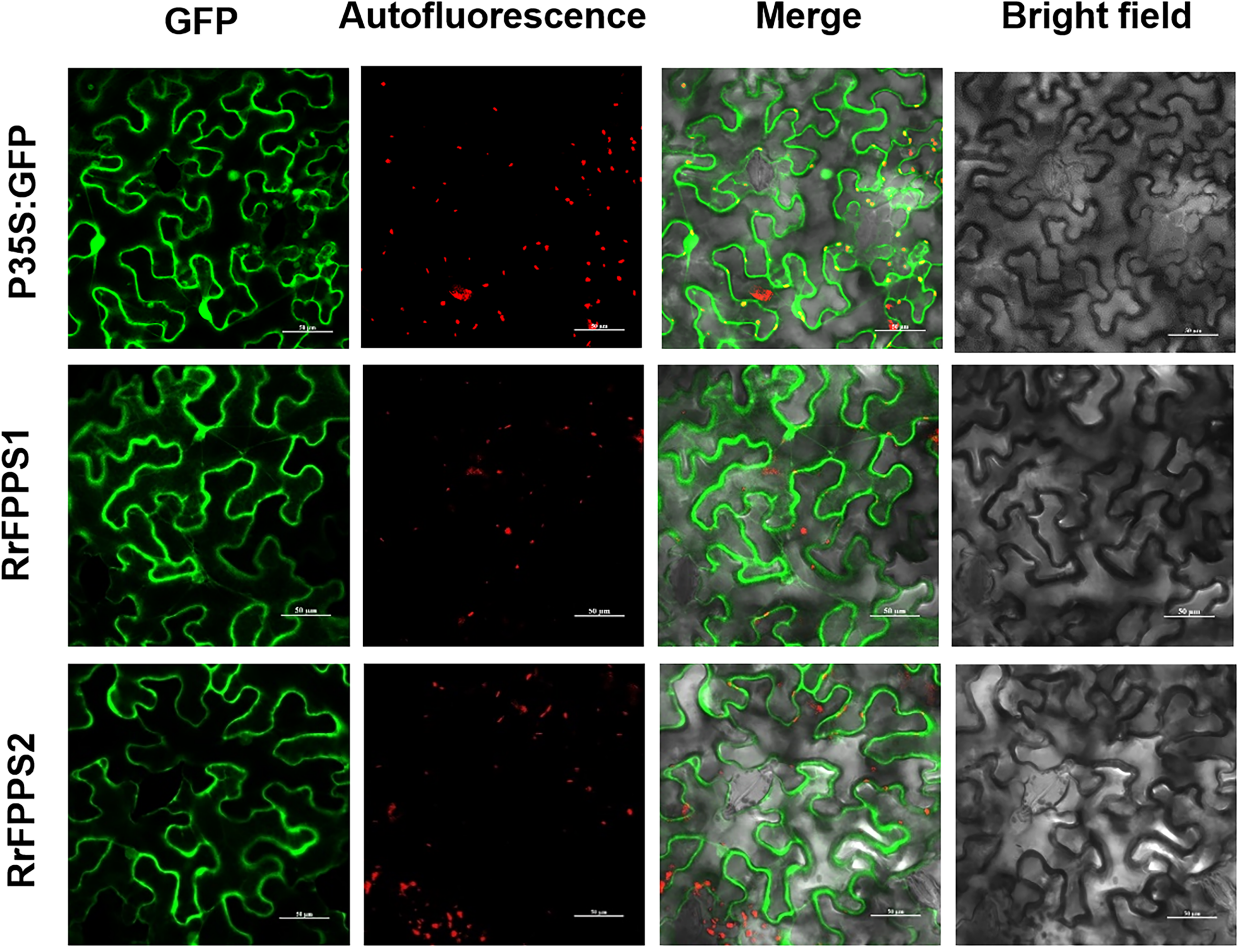
Subcellular localization of RrFPPS1 and RrFPPS2 in the *N. benthamiana* leaves. GFP (green fluorescent protein) signals from the adaxial leaf surface were observed by using a confocal laser scanning microscope. ‘GFP’ showed the green signal of the RrFPPSs–GFP fusion proteins or control (the GFP protein was expressed by the pCAMBIA1300-cGFP empty vector as control); ‘Autofluorescence’ showed the red autofluorescence of chlorophyll; ‘Merge’ showed the overlay of GFP and autofluorescence and ‘Bright field’ showed the bright field photograph.

FPP is commonly synthesized through the cytosolic MVA pathway (Chen et al., 2011; Sapir-Mir et al., 2008). To investigate the subcellular localization of RrFPPSs, we expressed them in *N. benthamiana* leaves. The results indicated that RrFPPS1 and RrFPPS2 were localized in the cytosol (Fig. 4). These findings support the notion that RrFPPS1 is responsible for producing FPP in the cytosol, which is then used in the biosynthesis of FPP-derived compounds.

### Functional characterization of the recombinant RrFPPS1

**Figure 5:**
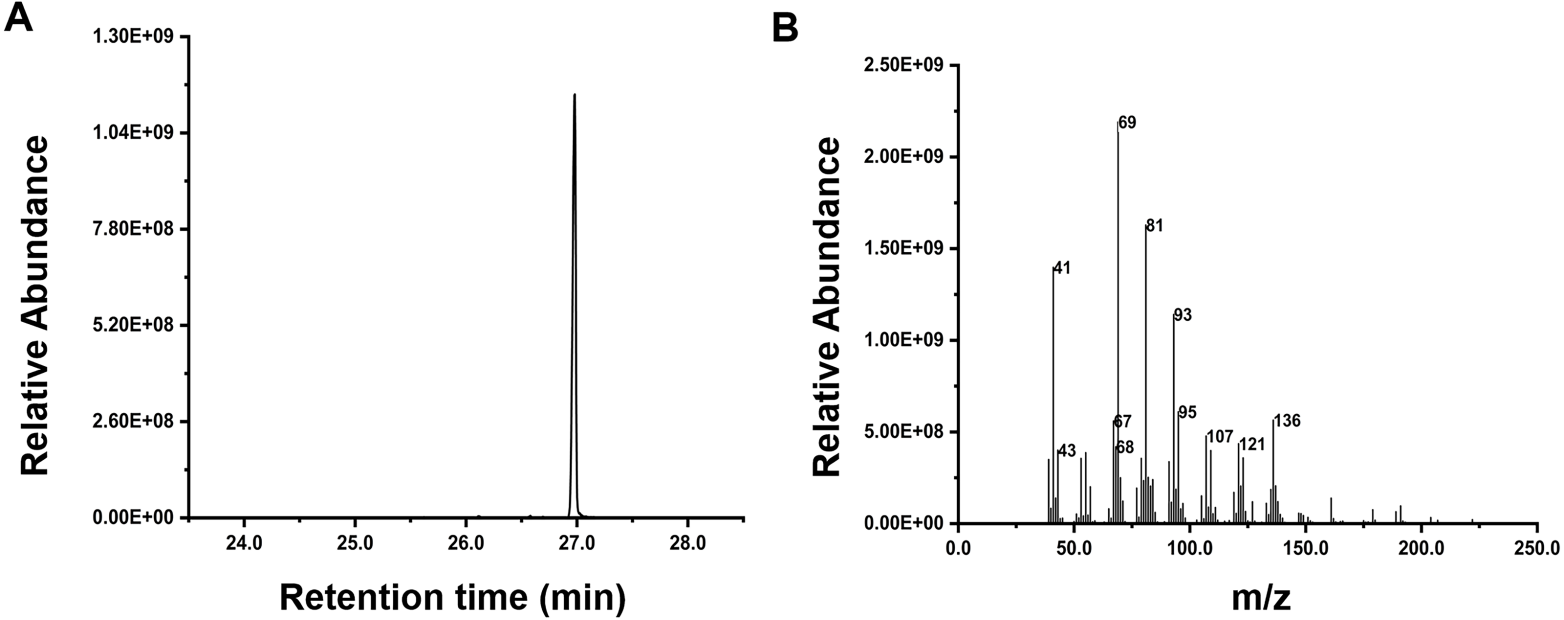
GC-MS analysis of the reaction products catalyzed by purified recombinant RrFPPS1 incubated with IPP and DMAPP. (A) The reaction products catalyzed by recombinant RrFPPS1 using IPP and DMAPP as substrates. (B) The mass spectrogram of the reaction products catalyzed by recombinant RrFPPS1.

Inspired by the discovery of the bifunctionality of RcG/FPPS1 from *R. chinensis*, which produces GPP and FPP in the cytosolic MVA pathway, we investigated the function of RrFPPS1. To elucidate the role of RrFPPS1, we expressed and purified the recombinant protein in *E. coli* using Ni-NTA chromatography. The RrFPPS1 protein was subjected to *in vitro* enzyme activity assays using DMAPP and IPP as substrates. GC-MS for product analysis conclusively demonstrated the function of RrFPPS1 as a fully functional FPP synthase (Fig. 5).

### Expression analysis of *RrFPPS* genes under salt treatment

**Figure 6:**
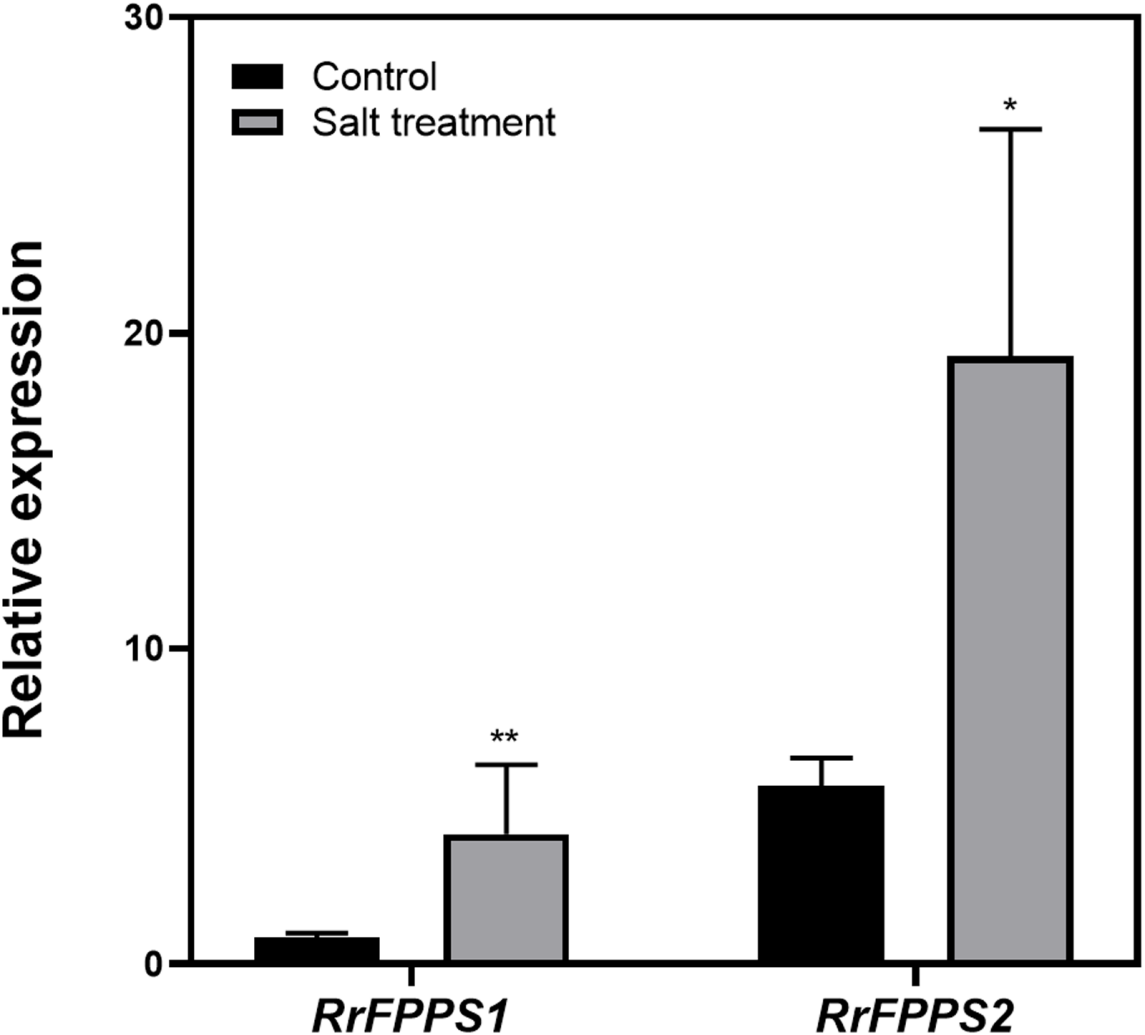
Change fold in *RrFPPSs* RNA levels in response to salt treatment. Expression changes of the *RrFPPSs* gene were determined in *R. rugosa* treated with salt in comparison with that in control cultures after the initiation of treatment: 1 h. Data are presented as mean ± SE, *n* = 3. Asterisks indicate statistically significant differences (*t*-test: *, *P* < 0.05; **, *P* < 0.01).

Terpenoids play crucial ecological roles in plant environment interactions. To investigate how *R. rugosa* responds to salt stress, we exposed *R. rugosa* plants to salt treatment and examined the expression levels of RrFPPS1 and RrFPPS2 using RT-qPCR. Our findings revealed a significant increase in the mRNA levels of RrFPPS1 and RrFPPS2 in *R. rugosa* leaves under salt treatments. This observation suggested a potential defensive role for these genes in response to salt-induced stress (Fig. 6).

## Discussion

This study comprehensively investigated the terpene biosynthesis in *R. rugosa*, focusing on FPP biosynthesis. The results led to the identification of RrFPPSs in the *R. rugosa* ‘Zi Zhi’ genome (J Wang, L Feng, 2022, unpublished data), representing a significant achievement in understanding the terpene biosynthetic pathway in this plant species. Tissue expression profiling revealed that RrFPPS1 was expressed in all tissues with distinct expression patterns. Subcellular localization of RrFPPS1 revealed that it is a cytosolic protein, and the phylogenetic analysis showed that RrFPPS1 is similar to RcFPPS2 (*R. chinensis*), suggesting potential functional similarities between these enzymes (Conart et al., 2023). Further examinations, such as biochemical assays or structural analysis, are needed to determine their biochemical functions in each species.

Although several FPPSs have been characterized from angiosperms and gymnosperms, identifying RrFPPS1 is a significant achievement because FPPS is a key enzyme involved in terpene biosynthesis in *R. rugosa*. The presence of conserved regions (FARM and SARM), which are typical of prenyltransferases, in RrFPPS1 indicated that these proteins are functionally active and may play a crucial role in FPPS-derived terpene biosynthesis in *R. rugosa*. The FARM and SARM regions are suggested to function as binding sites for the diphosphate moieties of IPP and allylic substrates (Adiwilaga & Kush, 1996) (Fig. 1). The FARM in domain II plays a role in the determination of chain length for the resulting prenyl pyrophosphates in the presence of a conserved Phe residue located five amino acids upstream of the DDxxD motif. The SARM in domain V is the binding site for the homoallylic substrate IPP (Ohnuma et al., 1996). Therefore, the conservation of these regions suggested that RrFPPS may share a similar mechanism of substrate binding and catalysis with other plant FPPS enzymes. However, this finding must be validated by biochemical experiments or structural similarity analysis. Phylogenetic analysis revealed that RrFPPS1 is highly similar to RcFPPS2 and falls into the angiosperm clade. The FPPS enzymes in gymnosperms may primarily contribute to metabolites such as sterols, which are also derived from FPP, while in angiosperms, apart from participating in primary metabolism, FPPs are likely to be involved in the volatile compounds found in flowers and belong to secondary metabolites (Weng et al., 2021). These findings suggested that RrFPPS1 shares a close genetic relationship with other FPPSs within the Rosaceae family, indicating the conserved function of FPPS in the Rosaceae family.

Tissue expression profiling of RrFPPS in *R. rugosa* provided valuable insights into the tissue-specific expression patterns of RrFPPS1. A preferential accumulation of RrFPPS1 mRNAs was observed in lateral branches, followed by flowers and roots. The significantly higher expression levels of RrFPPS1 in flowers compared with that in leaves suggested its essential role in the biosynthesis of terpenes, which are known to contribute to the enchanting flower scent that is characteristic of roses. Our study and the recent research by Conart et al. (2023), suggested that although RrFPPS1 plays a more dominant role in plant primary metabolism, RrFPPS2 is more involved in floral scent biosynthesis (Weng et al., 2021). These findings indicated that RrFPPS1 possibly has an important role in the biosynthesis of other terpenoids, such as terpene alcohols, sterols, ubiquinone, photosynthetic pigments, and plant hormones, all of which are essential for plant growth, development, and immunity (Degenhardt, Kollner & Gershenzon, 2009). Furthermore, our study indicated that the expression of two *RrFPPS* genes significantly responded to salt treatment. We propose that the upregulation of the *RrFPPS* genes can increase FPP substrate availability for sesquiterpenoid and triterpenoid biosynthesis, which could aid in coping with challenging environments (Teuber et al., 2008). These findings underscored the ecological importance of RrFPPSs in *R. rugosa*’s adaptation to its habitat.

Functional characterization of the recombinant RrFPPS1 protein revealed that it can produce FPP from IPP and DMAPP substrates, indicating that it is functionally active and plays a crucial role in the synthesis of important secondary metabolites. In particular, sesquiterpenes, are responsible for imparting pungent or aromatic flavors to specific plant tissues and are released in response to wounding and insect attack (Rosenkranz et al., 2021). In roses, sesquiterpenes have been traditionally used as flavors and fragrances and possess various biological properties, such as anticancer and antimalarial activities (Zhou et al., 2020).

Despite the progress made in this study, its limitations must be acknowledged. For fully elucidating the specific roles and functions of the *RrFPPS*1 gene, virus-induced gene silencing (VIGS) or CRISPR-Cas9 techniques may be necessary to precisely manipulate *RrFPPS1* expression. These approaches would provide comprehensive insights into *R. rugosa*. With this additional information at our disposal, we can enhance our comprehension of the mechanisms governing terpene biosynthesis and potentially explore novel targets for genetic modification or breeding programs in the future.

## Conclusions

The cloned and characterized of FPPSs from *R. rugosa* reported in this study are involved in terpene biosynthesis. The relative expression of RrFPPS1 was the highest in the flowers, and that of RrFPPS2 was the highest in the branch. Subcellular localization analysis revealed that RrFPPS1 and RrFPPS2 were cytoplasmic. Our study fills a gap in the understanding of the molecular mechanisms involved in sesquiterpene biosynthesis in *R. rugosa* and its function response to salt stress. The findings opened new avenues for the overexpression of the native *FPPS* gene into *R. rugosa*.

## Supplemental Information

10.7717/peerj.16929/supp-1 Supplemental Information 1 Raw data

10.7717/peerj.16929/supp-2 Supplemental Information 2 MIQE checklist

10.7717/peerj.16929/supp-3 Supplemental Information 3 List of related primers and probe sequences used in this study

10.7717/peerj.16929/supp-4 Supplemental Information 4 Protein sequences used for phylogenetic analysis in this study

## Additional Information and Declarations

### Data Availability

The following information was supplied regarding data availability: The raw measurements are available in the Supplemental Files.
