# Supplementary material for: Molecular cloning and characterization of farnesyl diphosphate synthase from Rosa rugosa Thunb associated with salinity stress
Source: PeerJ. 2024 Feb 29;12:e16929. doi: 10.7717/peerj.16929 (PMC10909355; doi:10.7717/peerj.16929)
Supplement: Supplemental Information 3 [file peerj-12-16929-s003.docx]

| Gene | Primers | Note |
| --- | --- | --- |
| *RrFPPS1* | Forward: ATGAGCAATTTAAGAGCCAAGTTC  Reverse: ATGACTCTCTTGATTTTC | CDS cloning |
| *RrFPPS2* | Forward : ATGGCGGATCTCAAGTCAAAG  Reverse :CTACTTTTTCCTCTTGTAAATCTTACCC | CDS cloning |
| *RrFPPS1* | Forward : AGTCTTGTGCACTTGGTTGGTGT  Reverse : CTGAACCAGCAGGGCTGACC | RT-qPCR |
| *RrFPPS2* | Forward : TCTTCCAAGCCAGCGCTCTT  Reverse : GCCGTGTGTGAGAGCCATCC | RT-qPCR |
| *5.8S* | Forward : CGGCAACGGATATCTCGG  Reverse : TGTGACGCCCAGGCAGACG | RT-qPCR (reference) |
| *RrFPPS1* | Forward :gagaacacgggggacgagctcATGAGCAATTTAAGAGCCAAG  Reverse : gctcaccatgtcgactctagaCTTCTGCCTCTTGTATATTTTTGC | Subcellular localization |
| *RrFPPS2* | Forward :gagaacacgggggacgagctcATGGCGGATCTCAAGTCAAAG  Reverse :gctcaccatgtcgactctagaCTTTTTCCTCTTGTAAATCTTACCC | Subcellular localization |

**List of related primers and probe sequences used in this study**
