# Supplementary material for: Molecular cloning and characterization of farnesyl diphosphate synthase from Rosa rugosa Thunb associated with salinity stress
Source: PeerJ. 2024 Feb 29;12:e16929. doi: 10.7717/peerj.16929 (PMC10909355; doi:10.7717/peerj.16929)
Supplement: Supplemental Information 4 [file peerj-12-16929-s004.docx]

**Protein sequences used for phylogenetic analysis in this study**

**>*Rosa rugosa* FPPS1 (AKT74338.1)**

MSNLRAKFMEVYSVLKSELINDPAFEFTDVSRQWIERMLDYNVPGGKLNRGLSVVDSFKLLKEGRELTDEEVFQSCALGWCIEWLQAYFLVLDDIMDGSHTRRGQPCWFRQPKIGMIAANDGIILRNHIPRILKNHFRVMPYYVDLLDLFNEVEFQTAHGQMIDLITTHEGEKDLSKYSLEIHRRIVQYKTAYYSFYLPVACALVMAGENVESHADVKNILIEMGTYFQVQDDYLDCFGDPEVIGKVGTDIQDFKCSWMVVKALELSNEEQKKLLHENYGKDDQECIAKVKELYNVLDLQGVFAEYESSSYDKLTKSIEAHPSKAVQAVLKSFLAKIYKRQK

**>*Rosa rugosa* FPPS2/RcG/FPPS (A0A2P6Q231)**

MADLKSKFLNVYSVLKSELLEDPAFEFTDASRQWVERMLDYNVPGGKLNRGLSVIDSYQLLQQGRELTEDEIFQASALGWCIEWLQAFFLVLDDMMDGSHTRRGQPCWFRLPKVGLIAANDGVLLRNHIPRILKKHFRQKPYYVDLVDLFNEVEFQTASGQLIDLITTIDGEKDLSKYSLSIHRRIVQYKTAYYSFYLSVACALLMSGEELDKHIDVKNLLVDMGIYFQVQDDYLDCFGDPETIGKIGTDIEDFKCSWLVVKALELSNEEQKKILHENYGNPDPAKVAKVKALYKELDLQGVFAEYERQSYEKLISSIEAHPSKAVQEVLKSFLGKIYKRKK

**>*Fragaria vesca* FPPS (XP_004294906.1)**

msnlkvkflevysvlkselindpafeftdvsrqwiermldynvpggklnrglsvvdslkllkeggeltddevfqscalgwciewlqayflvlddimdgshtrrgqpcwfrlpkigmiaandgiilrnhiprilkkhfrvkpyyvdlldlfnevefqtahgqmidlitthegekdlskyslgihrrivqyktayysfylpvacallmagenleshadvknvlvemgtyfqvqddyldcfgdpevigkvgtdiqdfkcswlvvkalelsneeqkkllhenygkddqeciakvkelynaldlqgvfaeyesssydkitksieahpskavqavlksflakiykrlk

**>*Malus domestica* FPPS (AAM08927.1)**

madlkskflkvysvlkselledpafdftndsrqwvermldynvpggklnrglsvidsyqllqqgreltedeiflasalgwciewlqafflvlddimdgshtrrgqpcwfrlpkvgmiavndgvvlrnhiprilrkyfrekpyyvdlldlfnevefqtasgqmidlittiegekdlskyslsihrrivqyktayysfylsvacallmsgeelekhidvknilvemgiyfqvqddyldcfgdpetigkigtdiedfkcswlvvkalelcneeqkkvlhenygkpdpenvatvkalykeldiegvfadyesksykkltswieghpskavqsvlksflgkiykrqk

**>*Centella asiatica* FPPS (AAV58896.1)**

msdlktrflevysvlksdllndpafeftddsrqwvermldynvpggklnrglsvidsykllkegkelsddeivlssalgwciewlqayflvlddimdgshtrrgqpcwfripkvgmiaindgillrnhiprilkkhfrqkpyyvdlldlfnevefqtacgqmidlittlvgekdlskyslpihrrivqyktayysfylpvacallmagedlekhtnvkdiliemgtyfqvqddyldcfgapevigkigtdiedfkcswlvvkalelsneeqkkclhenygkedpacvakikelykdlklqdvfaeyesksyeklikfieahpnqsvqavlksflgkiykrqk

**>*Lupinus albus* FPPS (P49351.1)**

madlrstflnvysvlksellhdpafefspdsrqwldrmldynvpggklnrglsvidsyrllkdghelnddeiflasalgwciewlqayflvlddimdnshtrrgqpcwfrvpkvgmiaandgvllrnhiprilkkhfrgkpyyadlldlfnevefqtasgqmidlittlegekdlskytlslhrrivqyktayysfylpvacallmvgenldnhidvknilvdmgtyfqvqddyldcfgapetigkigtdiedfkcswlvvkalelsndeqkkvlydnygkpdpanvakvkalydelnlqgvfteyesksyeklvtsieahpskavqallksflgkiykrqk

**>*Humulus lupulus* FPPS (BAB40665.1)**

msglrskfmevysilksellndpafeftddsrqwvermldynvpggklnrglsvidsyqllkggkelteeeifltsalgwciewlqayflvlddimdnsvtrrgqpcwfrvpkvgliaandgillrnhiprilkkhfkgksyyvdlldlfnevefqtasgqmidlittiegekdlskysiplhhrivqyktayysfylpvacalvmagenldnhvdvknvliemgtyfqvqddyldcfghpdvigkigtdiedfkcswlvvkaleiateeqkkmlfehygkgdeasvkkvkelykaldlegvfadyenasyqkliksieahpkeevqavlksflakiykrqk

**>*Panax ginseng* FPPS (AAY87903.1)**

msdlktrflevysvlksellndpafeftddsrqwvermldynvpggklnrglsvidsykllkegkelsddeiflssalgwciewlqayflvlddimdsshtrrgqpcwfrlpkvgmiavndgillrnhiprilkkhfrqkpyyvdlldlfnevefqtasgqmidlittlvgekdlskyslpihrrivqyktayysfylpvacallmsgedlekhtnvkdiliemgtyfqvqddyldcfgapevigkigtdiedfkcswlvvkalelsneeqkkflhenygkddpasvakvkelyntlklqdvfaeyesksydklikfieahpsqavqavlksflgkiykrqk

**>*Prunus persica* FPPS (XP_007211529.1)**

madlkskflqvysvlkselledpafeftndsrqwvermmdynvpggklnrglsvidsyqllqegreltedeiflasalgwciewlqafflvlddimdgshtrrgqpcwfrlpkvgmiavndgvvlrnhiprilrkhfrekpyyvdlldlfnevefqtasgqmidlittiqgekdlskyslsihrrivqyktayysfylsvacallmsgeelenhidvknlliemgiyfqvqddyldcygnpetigkigtdiedfkcswlvvkalelsneeqkkilyenygkadpenvakvkalykeldiegafadyesksyekltsgieghpskavqavlksflgkiykrkk

**>*Vitis vinifera* FPPS (AAX76910.1)**

msetkskflevysvlksellndpafeftddsrqwvermldynvpggklnrglsvvdsykllqgrqltddevflacvlglciewlqayflvlddimdnshtrrgqpcwfrvpkvgmiaandgvilrnqiprilknhfkgkpyyvdlldlfnevefqtasgqmidlittiegekdlskyslplhrrivqyktayysfhlpvacallmagenldnhtsvkdilvqmgiyfqvqddyldcfgdpqvigkigtdiedfkcswlivkaleicneeqkktlygnygkadpanvakvkalykdldlqgvfleyesksyetlvssieahpskavqavlksflgkiykrqk

**>*Potentilla anserina* FPPS (XP_050367607.1)**

mvdlrskflnvysvlksellqdpafeftdtsrqwveqmldynvpggklnrglsvidsyqllqqgreltedeifqasalgwciewlqafflvlddimdgshtrrgqpcwfrlpkvgliaandgvvlrnhiprilkkyfrqkpyyvdlldlfnevefqtasgqmidlittidgekdlskyslavhrrivqyktayysfylsvacallmsgealekhidvknllidmgiyfqvqddyldcfgdpetigkigtdiedfkcswlvvkalelsneeqkktlhenygnpdpakvarvkalykeldiqgvfaeyerqsyeklissieahpskavqevlksflgkiykrqk

**>*Hevea brasiliensis* FPPS (AY135188)**

madlkstflkvysvlkqelledpafewtpdsrqwvermldynvpggklnrglsvidsykllkegqelteeeiflasalgwciewlqayflvlddimdsshtrrgqpcwfrvpkvgliaandgillrnhiprilkkhfrgkayyvdlldlfnevefqtasgqmidlittlegekdlskytlslhrrivqyktayysfylpvacalliagenldnhivvkdilvqmgiyfqvqddyldcfgdpetigkigtdiedfkcswlvvkalelcneeqkkvlyehygkadpasvakvkvlynelklqgvfteyenesykklvtsiea hpskpvqavlksflakiykrqk

**>*Artemisia annua* FPPS (AAD17204)**

mssidlkskflkvydtlkselindpafefdddsrqwiekmldynvpggklnrglsvvdsyqllkggelsddeiflssalgwciewlqayflvlddimdeshtrrgqpcwfrllkvgmiaandgillrnhvprilkkhfrgkpyyvdlvdlfnevefqtasgqmidlittlvgekdlskyslsihrrivqyktayysfylpvacallmfgedldkhvevknmlvemgtyfqvqddyldcfgapevigkigtdiedfkcswlvvkalelaneeqkkvlhenygkkdpasvakvkevyhtlnlqavfedyeatsykklitsienrpskavqavlksflgkiykrqk

**>*Oryza sativa* FPPS (O04882)**

maaavvangasgdsskaafaeiysrlkeemledpafeftdeslqwidrmldynvlggkcnrgisvidsfkmlkgtdvlnkeetflactlgwciewlqayflvlddimdnsqtrrgqpcwfrvpqvgliavndgiilrnhisrilqrhfkgklyyvdlidlfnevefktasgqlldlitthegekdltkynltvhrrivqyktayysfylpvacalllsgenldnfgdvknilvemgtyfqvqddyldcygdpefigkigtdiedykcswlvvqaleradenqkhilfenygkpdpecvakvkdlykelnleavfheyeresynkliadieahpnkavqnvlksflhkiykrqk

**>*Zea mays* FPPS (P49353.1)**

maaggngaggdtraafariyktlkeelltdpafefteesrqwidrmvdynvlggkcnrglsvvdsykllkgadalgeeetflactlgwciewlqafflvlddimddshtrrgqpcwfrvpqvgliaandgiilrnhisrilrrhfkgkpyyadlldlfnevefktasgqlldlitthegekdltkynitvhgrivqyktayysfylpvacalllsgenldnygdvenilvemgtyfqvqddyldcygdpefigkigtdiedykcswlvvqaleradesqkrilfenygkkdpacvakvknlykeldleavfqeyenesykkliadieaqpsiavqkvlksflhkiykrqk

**>*Santalum album* FPPS (ADO87007.1)**

mgdrktkfleaysvlksellrdpafnftdasrqwvdrmldynvpggklnrglsvidsyellkegkeltddeiflasalgwciewlqayflvlddimdgshtrrgqpcwfrlpevgliavndgimlrnhiprilkkhfknkpyyvelldlfnevefqttsgqmidlittlegqkdlskysmpihhrivqyktayysfylpvacallmsgenldshtevekilvemgtyfqvqddyldcfghpdvigkigtdiedfkcswlvvkalelsneeqkkllyenygkadeasvakvkalykeldlegafveyenasyekiissievqpskavqavlksflakiykrqk

**>*Paeonia lactiflora* FPPS (AKJ26301.1)**

madvngtrmdlrskflnvysvlksellddpafeftnesrqwvermldynvpggklnrglsvidsyqilkdgkelteeeifltsalgwciewlqayflvlddimdgshtrrgqpcwfrlpkvgliaandgiilrnhiprilknhfrersyyvdlldlfnevefqtasgqmidlittlegekdlskyslslhrrivqfktayysfylpvacalvmsgenldnhvavkdilvemgtyfqvqddyldcygepekigkigtdiedfkcswmvvkalelcneeqkkilhehygkadpadvakvkalyneldlqgvfaeyenksyeklitsieahpskavqavlksflgkiykrqk

**>*Jasminum sambac* FPPS (AIY24421)**

manqngansdlrtkflevysvlksellndpafewtdnsrqwvermldynvpggklnrglsvidsykllkegkelteeeiflasalgwciewlqayflvlddimdnshtrrgqpcwfrvekvgmiaandgiilrnhiprilrkhfrdkpyyvdlldlfnevefqtasgqmidlittiegekdlskyslslhrrivqyktayysfyipvacallmanenldkhidvknilidmgiyfqvqddyldcfgepekigkigtdiedfkcswlvvkalelcneeqkkilfehygkddpadvakikavyndinlqgvyeefenksyekltssieahpskavqavlksflgkiyrrqk

**>*Picea abies* FPPS (ACA21460.1)**

MASNGIVDVKTKFEEIYLELKAQILNDPAFDYTEDARQWVEKMLDYTVPGGKLNRGLSVIDSYRLLKAGKEISEDEVFLGCVLGWCIEWLQAYFLILDDIMDSSHTRRGQPCWFRLPKVGLIAVNDGILLRNHICRILKKHFRTKPYYVDLLDLFNEVEFQTASGQLLDLITTHEGATDLSKYKMPTYVRIVQYKTAYYSFYLPVACALVMAGENLDNHVDVKNILVEMGTYFQVQDDYLDCFGDPEVIGKIGTDIEDFKCSWLVVQALERANESQLQRLYANYGKKDPSCVAEVKAVYRDLGLQDVFLEYERTSHKELISSIEAQENESLQLVLKSFLGKIYKRQK

**>*Ginkgo biloba* FPPS (AKJ26301.1)**

mqfpslrklhsifrvealyygiglsgrestskefrslhpgfaamesncnantrskflevynvlksqilndsafqctddarqwiekmldytvpggklnrglsvidsyrllktgkeitedevflgcvlgwciewlqayflvlddimdgshtrrgqpcwfrlpqvgliaandgillrthisrilklhfqtkpyyvdlcdlfnevefqtasgqmldlitthegaidlakykmptylrivqyktayysfylpvacallmagenldnfvavknilvqmgtyfqvqddyldcfgdpevigkigtdiedfkcswlivqaleranesqrkqlydnygkadpscvaavkaiyrdlgiqdifleyersshkelissieaqenesvqlvlksflgkiykrqk

**>*Rosa chinensis* FPPS2 (A0A2P6QLH7)**

msnlrakfmevysvlkselindpafeftdvsrqwiermldynvpggklnrglsvvdsfkllkegreltdeevfqscalgwciewlqayflvlddimdgshtrrgqpcwfrqpkigmiaandgiilrnhiprilknhfrvmpyyvdlldlfnevefqtahgqmidlitthegekdlskysleihrrivqyktayysfylpvacalvmagknveshadvkniliemgtyfqvqddyldcfgdpevigkvgtdiqdfkcswmvvkalelsneeqkkllhenygkddqeciakvkelynvldlqgvfteyesssydkltksieahpskavqavlksflakiykrqk
